# Characterizing inflammatory biomarkers in post-stroke seizure risk and outcome prognostication

**DOI:** 10.1371/journal.pone.0345752

**Published:** 2026-03-31

**Authors:** Ethan Y. Wang, Shubham Misra, Jennifer Yan, Pei Yi Chook, Yuki Kawamura, Rachel Kitagawa, Jennifer A. Kim, Emily J. Gilmore, Adam de Havenon, Adithya Sivaraju, Lawrence J. Hirsch, Guido J. Falcone, Srikant Rangaraju, Lauren H. Sansing, Jessica Magid-Bernstein, Nishant K. Mishra

**Affiliations:** 1 Department of Neurology, Yale School of Medicine, New Haven, Connecticut, United States of America; 2 Universiti Malaya, Kuala Lumpur, Malaysia,; 3 University of Cambridge, Cambridge, United Kingdom; University of Modena and Reggio Emilia, ITALY

## Abstract

Post-stroke seizures (PSS) are sequelae of intracerebral hemorrhage (ICH) that may negatively impact patient outcomes. Available literature suggests that inflammatory biomarkers contribute to epileptogenesis as well as mortality. This retrospective cohort study aimed to elucidate the prognostic ability of CCL2, IL6, and IL8 in PSS development. ICH data were collected at Yale New Haven Hospital from 2014 to 2021. Plasma biomarker levels were measured via cytometric bead array. Patients with EEG-recorded epileptiform discharges, EEG-recorded seizures, or clinical seizures after ICH were defined as seizures and epileptiform discharges (SED), while those without were defined as non-SED. SED was further divided into early and late, occurring before and 7 days post-ICH, respectively. Additionally, we examined if biomarkers were associated with poor outcome (modified Rankin Scale score of 3–6) and mortality at 90, 180, and 365 days post-ICH. We conducted univariable and multivariable logistic regression analyses and reported the findings as Odds Ratio (OR) and 95% CI. We examined 172 patients with ICH, of whom 33 had early SED, 29 had late SED, and 110 had no SED. In univariable analyses, CCL2, ICH volume, diabetes, and lobar ICH were significantly associated with late SED, whereas NIHSS score at admission, ICH volume, and lobar ICH were significantly associated with early SED (p < 0.05). Lower CCL2 levels were independent predictors of late SED in multivariable analyses (OR 0.58; 95% CI 0.41–0.80, p < 0.001), but not of early SED. Additionally, we identified an independent association between higher CCL2 levels and 90-day mortality in multivariable analysis for the combined early and late SED cohorts (OR 1.87; 95% CI 1.03–3.37, p = 0.038). Additional studies investigating additional aspects of biomarkers, such as their temporal profile post-stroke within 24 hours or beyond 72 hours, are needed.

## Introduction

Seizures are potential complications in patients who experience intracerebral hemorrhages (ICH), which can adversely affect their health outcomes [1,2]. These seizures, or post-stroke seizures (PSS), are divided into early PSS, which occur within seven days of stroke occurrence, and late PSS, which occur after seven days [3,4]. While their mechanisms likely exist within a shared spectrum [3,5], early seizures are understood to mainly result from acute inflammation due to insult, whereas late seizures likely result from longer-lasting tissue disruption, which can have chronic consequences like post-stroke epilepsy (PSE) [6]. Regardless of the timing of PSS, there is evidence of a high mortality risk by ten years [7].

Due to the disparate outcomes in PSS patients, there is a need to enhance our ability to identify patients at risk of PSS and to elucidate underlying biological mechanisms. This is especially true for late seizures, as they may signal chronic illness and morbidity such as PSE. Besides those with overt PSS, there is also evidence that patients with EEG-captured epileptiform discharges have an increased risk of PSS in ischemic and hemorrhagic stroke, possibly reflecting increased cortical hyperexcitability contributing to epileptogenesis [8–11]. By identifying risk factors leading to seizures and epileptiform discharges (SED) post-stroke, we can better identify the patient population that could benefit from potential therapeutics and other interventions.

Existing risk factors for PSS development have been reported in this pursuit. The most well-known are the cortical location of the lesion, younger patient age, hematoma size, and presence of early seizures [3,12,13]. However, additional factors may also predispose patients to experiencing PSS. Recent research has pointed to biomarkers, such as interleukin (IL)-6 and IL-1β, as possible predictors [14]. This is likely due to their involvement in inflammatory responses, alteration of the blood-brain barrier, and other mechanisms of seizure initiation and propagation in post-stroke patients [15–17].

## Objectives

We conducted this retrospective cohort study to:

1. Determine whether inflammatory plasma biomarkers are independently associated with SED development, with a focus on late SED.
2. Determine whether inflammatory plasma biomarkers are predictors of poor functional outcome or mortality in late SED patients at 90, 180, or 365 days post-stroke.

## Methods

### Study sample

This was a retrospective study of data of ICH patients admitted to the Yale New Haven Hospital system from 2014 to 2021. Data were accessed on 18^th^ July 2023, and all data were anonymized before access. Patients were included if they were over the age of 18 years, had a diagnosis of ICH confirmed by neuroimaging, and had biomarker data collected during the first 24–72 hours of admission. Biomarkers included monocyte chemoattractant protein-1 (CCL2), IL6, and CXCL8 (IL8). We excluded patients with ischemic stroke/transient ischemic attack (TIA) and patients with a history of cocaine use, seizures, or epilepsy before ICH.

Patients were classified as SED if they had a documented history of clinical seizures, EEG-recorded seizures, or EEG-recorded epileptiform discharges during and after hospitalization. These SED patients were further classified as early or late. Early is defined as SED occurring within 7 days after ICH, while late is defined as 7 or more days after ICH. Those who did not have these findings were deemed non-SED. Early and late SED patients were also divided based on poor functional outcomes defined as modified Rankin Scale (mRS) scores of 3–6 and mortality at 90-, 180-, and 365-day follow-up. Consent was collected verbally or through writing, which was documented in the patient’s electronic medical record.

### Sample collection and biomarker measurement

Plasma samples were collected and analyzed from ICH patients within 24 and 72 hours of hospital admission from 15^th^ August 2014–28^th^ July 2021. The biomarker levels in plasma were measured using a cytometric bead array at 24 hours or 72 hours after admission [18]. For patients with both 24-hour and 72-hour biomarker data (38/172), biomarker values were averaged, as values were not significantly different between timepoints (see Table S1 in S1 File). The overall proportion of patients with samples collected at 24 hours, 72 hours, or both is included in Table S2 in S1 File.

### Data collection

Patient characteristics were collected through their electronic health records, including demographics (age, gender, race, body mass index), vascular risk factors (hypertension, diabetes, hyperlipidemia, atrial fibrillation, coagulopathy, coronary artery disease, prior TIA or myocardial infarction), clinical scores (National Institutes of Health Stroke Scale (NIHSS), ICH score), radiological parameters (ICH volume, ICH type), and inflammatory biomarker levels. Functional outcomes and mortality at 90-, 180-, and 365-day follow-up were also collected.

### Outcomes

Our primary outcome was the prognostic value of inflammatory biomarkers in predicting early and late SED development. Our secondary outcome was the prognostic value of inflammatory biomarkers in predicting poor functional outcome and mortality in early, late, and early + late SED patients at 90-, 180- and 365-day follow-up.

### Ethics approval

The plasma samples were collected under IRB 1405014045 and analyzed under IRB 1506016023, both through approval by the Institutional Review Board of Yale University.

### Statistical analysis

The data normality was checked using the Shapiro-Wilk test. Biomarker values were log-transformed. Biomarkers with ≤50% values below the limit of detection were imputed from a normal distribution using Perseus 2.0.11. We conducted univariable analysis for early and late SED using Mann-Whitney U tests for continuous variables except the log-transformed biomarkers, which were analyzed via independent-sample t-tests, and the Pearson chi-square test for categorical variables.

Variables significant in univariable analysis (p < 0.05) were included in a backward stepwise multivariable logistic regression analysis to assess the independent association of biomarkers with early or late SED. We considered p-values <0.05 as statistically significant in the multivariable analyses. We report Odds Ratio (OR) and 95% Confidence Intervals (CI). Additionally, we conducted univariable (p < 0.1) and multivariable (p < 0.05) logistic regression analyses to examine the association of biomarkers with poor functional outcomes and mortality in SED groups at 90, 180, and 365 days. Analyses were done using SPSS (Version 28.0, IBM Corp, Armonk, NY) and STATA (Version 18, Stata Corp, College Station, TX).

## Results

We included 172 ICH patients, 33 with early SED, 29 with late SED, and 110 with non-SED. Baseline characteristics of all groups with p-values of univariable analyses are shown in Table 1. All biomarkers had > 50% of values that were above the limit of detection before imputation during plasma sample analysis: 94% of CCL2 values, 60% of IL6 values, and 74% IL8 values were above the limit of detection. We classified patients into two groups based on SED subtypes: early SED (N = 33) vs. late + non-SED (N = 139) and late SED (N = 29) vs. non-SED (N = 110) to identify predictors of early and late SED, respectively.

**Table 1:** Baseline Characteristics of ICH patients included in the study.

Characteristics	Early SED (N = 33)	Late SED (N = 29)	No SED (N = 110)	Early SED vs. Late + Non- SED (p-value)	Late SED vs. Non- SED (p-value)
Demographics					
Age	66 (58-76)	68 (53-77)	68 (61-77)	0.46	0.65
Male Gender	15 (45.5)	14 (48.3)	66 (60)	0.21	0.26
Caucasian	24 (72.7)	20 (69.0)	88 (80)	0.54	0.20
History of Smoking	15 (45.5)	11 (37.9)	46 (41.8)	0.64	0.71
Alcohol Use	13 (39.4)	6 (20.7)	32 (29.1)	0.17	0.37
Vascular Risk Factors					
Hypertension	20 (60.6)	17 (58.6)	83 (75.5)	0.20	0.07
Diabetes	7 (21.2)	0 (0)	31 (28.2)	0.89	0.001
Hyperlipidemia	12 (36.4)	14 (48.3)	40 (36.4)	0.79	0.24
Atrial fibrillation	4 (12.1)	6 (20.7)	19 (17.3)	0.42	0.67
Coagulopathy	2 (6.1)	1 (3.4)	1 (1)	0.11	0.31
Coronary Artery Disease	3 (9.1)	3 (10.3)	18 (16.4)	0.37	0.42
Prior Transient Ischemic Attack	3 (9.1)	2 (6.9)	2 (1.8)	0.10	0.15
Prior Myocardial Infarction	0 (0)	1 (3.4)	3 (2.7)	0.32	0.84
Inpatient Antiplatelet Use	2 (6.1)	4 (13.8)	24 (21.8)	0.06	0.34
Clinical Scores					
NIHSS at Admission	17 (8-21)	9 (5-16)	9.5 (4-17)	0.005	0.80
ICH Score	2 (1-3)	1 (0-2)	1 (0-2)	0.13	0.36
Radiological parameters					
ICH volume	22.2 (8.4-47.8)	20 (9.5-35.6)	8.2 (3-19.4)	0.008	0.005
Lobar ICH	20 (60.6)	22 (75.9)	26 (23.2)	0.006	<0.001
Biomarkers					
CCL2	7.40 (6.10-8.28)	5.42 (4.67-7.23)	7.28 (6.25-7.93)	0.35	<0.001
IL6	4.34 (2.74-6.04)	4.13 (2.55-5.10)	3.72 (2.75-5.15)	0.33	0.63
CXCL8 (IL8)	4.03 (3.50-4.69)	3.61 (3.11-4.82)	3.91 (3.25-4.58)	0.25	0.98

All values are in Median (IQR) or n (%). p-values from Mann-Whitney U test, independent t-test, or χ^2^ test.

**Predictors of early SED:** In the early SED group, NIHSS score at admission, ICH volume, and lobar ICH were significant in the univariable analysis (p < 0.05), whereas no biomarkers were associated with early SED (Table 1). Lobar ICH (OR 3.50; 95% CI 1.54–7.98) and NIHSS score at admission (OR 1.08; 95% CI 1.03–1.13) were independent predictors of early SED compared to late + non-SED in the multivariable analysis (Table 2).

**Table 2:** Multivariable prediction models for prognosticating Early SED and Late SED.

Predictor Variables	OR (95% CI)	P-value
**Prediction Model: Early SED**		
Lobar ICH	3.50 (1.54-7.98)	0.003
NIHSS Score at Admission	1.08 (1.03-1.13)	0.003
**Prediction Model: Late SED**		
Lobar ICH	10.5 (3.79-29.16)	<0.001
CCL2	0.58 (0.41-0.80)	<0.001

Early SED model: Adjusted for lobar ICH, NIHSS score at admission, and ICH volume. Late SED model: Adjusted for diabetes, lobar ICH, and CCL2 based on the lowest p-values to prevent model overfitting.

**Predictors of late SED:** In the late SED group, diabetes, ICH volume, lobar ICH, and CCL2 levels were significant in the univariable analysis (p < 0.05, Table 1). Lower CCL2 levels (OR 0.58; 95% CI 0.41–0.80) and lobar ICH (OR 10.5; 95% CI 3.79–29.16) were independent predictors of late SED compared to non-SED in the multivariable analysis (Table 2).

**Poor outcomes and mortality after early and late SED:** We identified age, diabetes, NIHSS score at admission, ICH score, ICH volume, CCL2, IL6, and IL8 as significant predictors of 90-day mortality in the univariable analysis (p < 0.1, Table 3). Higher CCL2 levels (OR 1.87; 95% CI 1.03–3.37) and NIHSS score at admission (OR 1.41; 95% CI 1.13–1.76) were independent predictors of 90-day mortality in patients with early and late SED combined after multivariable analysis (Table 4). A similar analysis was conducted to assess mortality and poor outcomes at 90, 180, and 365 days for patients with early SED, late SED, and early and late SED combined (apart from the 90-day mortality analysis of combined early and late SED described here), but no biomarker was found to be an independent predictor (Supplementary Tables S3-S10 in S1 File).

**Table 3:** Baseline Characteristics of Early and Late SED (Combined) by 90-Day Mortality.

Characteristics	90-Day Mortality (N = 16)	Non-90-Day Mortality (N = 46)	p-value
Demographics			
Age	72.5 (66.5-82.5)	66.0 (55.0-75.0)	0.03
Male Gender	7 (43.8)	22 (47.8)	0.78
Caucasian	11 (69.0)	33 (71.7)	0.82
History of Smoking	6 (37.5)	20 (43.5)	0.68
Alcohol Use	5 (31.3)	14 (30.4)	0.95
Vascular Risk Factors			
Hypertension	9 (56.3)	28 (60.9)	0.75
Diabetes	5 (31.3)	2 (4.35)	0.003
Hyperlipidemia	9 (56.3)	17 (37.0)	0.18
Atrial fibrillation	2 (12.5)	8 (17.4)	0.65
Coagulopathy	1 (6.3)	2 (4.4)	0.76
Coronary Artery Disease	2 (12.5)	4 (8.7)	0.66
Prior Transient Ischemic Attack	0 (0)	5 (10.9)	0.17
Prior Myocardial Infarction	0 (0)	1 (2.2)	0.55
Inpatient Antiplatelet Use	0 (0)	6 (13.0)	0.13
Clinical Scores			
NIHSS at Admission	20 (15-23)	9 (5-17)	0.0001
ICH Score	2.0 (1.5-3.0)	1.0 (0-2.0)	0.002
Radiological parameters			
ICH volume	35.7 (19.3-52.9)	17.7 (8.1-35.6)	0.02
Lobar ICH	10 (62.5)	32 (69.6)	0.60
Biomarkers			
CCL2	7.22 (5.91-8.23)	6.54 (4.75-7.92)	0.08
IL6	5.23 (3.38-6.16)	3.89 (2.55-5.13)	0.03
CXCL8 (IL8)	4.26 (3.75-5.48)	3.95 (3.01-4.69)	0.01

All values are in Median (IQR) or n (%). p-values from Mann-Whitney U test, independent t-test, or χ^2^ test

**Table 4:** Multivariable prediction model for 90-day mortality in Early and Late SED (Combined).

Predictor Variables	OR (95% CI)	P-value
**Prediction Model: Early SED + Late SED**		
CCL2	1.87 (1.03-3.37)	0.04
NIHSS at Admission	1.41 (1.13-1.76)	0.003

Model adjusted for CCL2, NIHSS at admission, IL6, age, ICH score, ICH volume, diabetes

## Discussion

Our study identified lobar ICH and lower CCL2 levels as independent predictors of late SED development. These findings support existing literature that having a lobar ICH may influence the development of seizures or epileptiform discharges after a hemorrhagic stroke. Many studies have illustrated the association between lobar hemorrhages and the development of seizures post-ICH [3,19–23]. The mechanism is thought to involve increased excitability of cortical neurons close to the lesion, triggering the likelihood of seizures [24,25]. The presence of cortical lesions can be used to help better identify patients who are at risk of PSS. Applications include serving as an enrichment criterion, like in the PEPSTEP trial, ensuring that potential therapies for PSS are tested on patients most likely to experience them [26].

CXCL8 (IL8) and IL6 have been associated with inflammation and seizures without directly examining post-hemorrhagic stroke seizures [27,28]. IL6 was also shown to be independently correlated with seizure recurrence in post-ischemic stroke patients in the cohort of Jia et al [29]. One major difference between ischemic stroke and hemorrhagic stroke is the presence of blood (and its various components) outside of the blood vessels, which can cause findings like cortical superficial siderosis in hemorrhagic stroke not seen in ischemic stroke [11].

CCL2 is a chemokine secreted by various immune cells, especially monocytes, to facilitate immune cell migration to areas of inflammation such as hemorrhagic lesions. Most literature supports its activity as a pro-inflammatory, epileptogenic biomarker [30–33]. CCL2 has also been linked to conditions such as childhood epilepsy and drug-resistant epilepsy [34,35]. Our findings appear to suggest that lower CCL2 levels are associated with late SED. Possible mechanisms include temporality-related effects, such that higher CCL2 levels within 24 hours may cause worsened outcomes, but between 24–72 hours of ICH may have protective effects against persistent hyperexcitability or lasting damage, causing late SEDs. Animal models have demonstrated that higher CCL2 levels or expression in ischemic stroke are associated with faster tissue recovery [36–38]. CCR2, the receptor for CCL2, inhibition leads to decreased anti-inflammatory response, increased mortality, and impaired behavior recovery in a mouse model, but after 72 hours post-insult [36,39]. We also identified an independent association of higher CCL2 levels and NIHSS score at admission with 90-day mortality in the combined early and late SED cohort. Our finding of increased 90-day mortality with higher CCL2 levels in the combined cohort may be reflective of a large vascular insult seen in the significance of the NIHSS score at admission for the early SED group and the combined SED (early and late) group. The insult could be significant to the point where lowered seizure and epileptiform discharge risk from possible neuroprotective effects is unable to compensate, leading to increased mortality at 90 days.

There are limitations to our study. The patient sample size was small (n = 172), including the early and late SED cohorts (n = 33 and n = 29, respectively) and selected through convenience sampling, decreasing the external validity and influencing our findings’ significance. EEGs were not collected on every patient, which may have led to undercounting of those with epileptiform discharges. We were also unable to verify that clinical seizures had corresponding changes on the EEG. The level of biomarkers in predicting outcomes may fluctuate and depend on the time it was collected, as differences between SED and non-SED groups may be in the temporal profile of the biomarkers themselves instead of differences in values at any given time. Research assessing the temporal profile of biomarkers in stroke has demonstrated many different variations. Most literature is limited to blood samples post-admission, leading to observation of broadly increasing/decreasing trends [40–42]. Ideally, biomarker levels are measured repeatedly, and frequently after the onset of symptoms, to closely assess temporal changes. This has led to findings of changes in trends within 24 hours in certain biomarkers [43].

Future research should include this distinction as well as serum samples at more frequent intervals to better track temporal trends of these biomarkers, especially CCL2. More studies should also focus on the effects of ICH specifically, given the differing mechanisms of ischemic stroke in causing hypoxia. Larger studies focused on patients not only with seizures, but post-stroke epilepsy are also needed to elucidate the relationship with inflammatory biomarkers. Future research could also incorporate current advancements in machine learning and AI algorithms. Current research in post-stroke seizure and epilepsy with machine learning has been done for both ischemic and hemorrhagic stroke, albeit with non-biomarker findings (NIHSS, cortical involvement) [44–46]. Our analysis relied on conventional methods, whereas the machine learning studies were able to use a larger sample of patients, which could greatly improve predictions and prognostication.

## Conclusion

In our retrospective analyses, we found that lower CCL2 levels were independent predictors of late SED. We also found an independent association of higher CCL2 levels in SED patients (early and late) with 90-day mortality. Additional studies investigating further aspects of biomarkers, such as levels within 24 hours or those past 72 hours, are needed.

## Supporting information

S1 File S1-10 Supplementary Tables (includes paired T-test for patients with 24- and 72-hour values and Timing of sample collection for all patient groups). (DOCX)

## References

[1] Vespa PM, O’Phelan K, Shah M, Mirabelli J, Starkman S, Kidwell C, et al. Acute seizures after intracerebral hemorrhage. Neurology. 2003;60(9):1441–6. doi: 10.1212/01.wnl.0000063316.47591.b4 12743228

[2] Misra S, Kasner SE, Dawson J, Tanaka T, Zhao Y, Zaveri HP, et al. Outcomes in Patients With Poststroke Seizures: A Systematic Review and Meta-Analysis. JAMA Neurol. 2023;80(11):1155–65. doi: 10.1001/jamaneurol.2023.3240 37721736 PMC10507596

[3] Haapaniemi E, Strbian D, Rossi C, Putaala J, Sipi T, Mustanoja S, et al. The CAVE Score for Predicting Late Seizures After Intracerebral Hemorrhage. Stroke. 2014;45(7):1971–6. 24876089 10.1161/STROKEAHA.114.004686

[4] Tanaka T, Ihara M, Fukuma K, Mishra NK, Koepp MJ, Guekht A. Pathophysiology, diagnosis, prognosis, and prevention of poststroke epilepsy: clinical and research implications. Neurology. 2024;102(11):e209450. doi: 10.1212/WNL.000000000000209450 PMC11175639 38759128

[5] Hsu C-J, Weng W-C, Peng SS-F, Lee W-T. Early-onset seizures are correlated with late-onset seizures in children with arterial ischemic stroke. Stroke. 2014;45(4):1161–3. doi: 10.1161/STROKEAHA.113.004015 24595587

[6] Camilo O, Goldstein LB. Seizures and epilepsy after ischemic stroke. Stroke. 2004;35(7):1769–75. doi: 10.1161/01.STR.0000130989.17100.96 15166395

[7] Hesdorffer DC, Benn EKT, Cascino GD, Hauser WA. Is a first acute symptomatic seizure epilepsy? Mortality and risk for recurrent seizure. Epilepsia. 2009;50(5):1102–8. doi: 10.1111/j.1528-1167.2008.01945.x 19374657

[8] Schubert KM, Dasari V, Oliveira AL, Tatillo C, Naeije G, Strzelczyk A. The Role of Electroencephalography in Predicting Post-Stroke Seizures and an Updated Prognostic Model (SeLECT-EEG). Ann Neurol. 2025. 10.1002/ana.27301 PMC12542305 40568812

[9] Punia V, Ellison L, Bena J, Chandan P, Sivaraju A, George P, et al. Acute epileptiform abnormalities are the primary predictors of post-stroke epilepsy: a matched, case-control study. Ann Clin Transl Neurol. 2022;9(4):558–63. doi: 10.1002/acn3.51534 35243824 PMC8994977

[10] Bentes C, Martins H, Peralta AR, Morgado C, Casimiro C, Franco AC, et al. Early EEG predicts poststroke epilepsy. Epilepsia Open. 2018;3(2):203–12. doi: 10.1002/epi4.12103 29881799 PMC5983181

[11] Tanaka T, Fukuma K, Abe S, Matsubara S, Ikeda S, Kamogawa N, et al. Association of Cortical Superficial Siderosis with Post-Stroke Epilepsy. Ann Neurol. 2023;93(2):357–70. doi: 10.1002/ana.26497 36053955 PMC10087209

[12] Kwon SY, Obeidat AZ, Sekar P, Moomaw CJ, Osborne J, Testai FD, et al. Risk factors for seizures after intracerebral hemorrhage: Ethnic/Racial Variations of Intracerebral Hemorrhage (ERICH) Study. Clin Neurol Neurosurg. 2020;192:105731. doi: 10.1016/j.clineuro.2020.105731 32062309 PMC7183434

[13] Zhang C, Wang X, Wang Y, Zhang J, Hu W, Ge M, et al. Risk factors for post-stroke seizures: a systematic review and meta-analysis. Epilepsy Res. 2014;108(10):1806–16. doi: 10.1016/j.eplepsyres.2014.09.030 25454500

[14] Misra S, Khan EI, Lam TT, Mazumder R, Gururangan K, Hickman LB. Common pathways of epileptogenesis in patients with epilepsy post–brain injury. Neurology. 2023;101(22):e2243–56. 10.1212/WNL.0000000000207749 PMC10727219 37550071

[15] Stellwagen D, Beattie EC, Seo JY, Malenka RC. Differential regulation of AMPA receptor and GABA receptor trafficking by tumor necrosis factor-alpha. J Neurosci. 2005;25(12):3219–28. doi: 10.1523/JNEUROSCI.4486-04.2005 15788779 PMC6725093

[16] Vezzani A, Balosso S, Ravizza T. Neuroinflammatory pathways as treatment targets and biomarkers in epilepsy. Nat Rev Neurol. 2019;15(8):459–72. doi: 10.1038/s41582-019-0217-x 31263255

[17] Abraira L, Giannini N, Santamarina E, Cazorla S, Bustamante A, Quintana M, et al. Correlation of blood biomarkers with early-onset seizures after an acute stroke event. Epilepsy Behav. 2020;104(Pt B):106549. doi: 10.1016/j.yebeh.2019.106549 31677998

[18] Morgan E, Varro R, Sepulveda H, Ember JA, Apgar J, Wilson J, et al. Cytometric bead array: a multiplexed assay platform with applications in various areas of biology. Clin Immunol. 2004;110(3):252–66. doi: 10.1016/j.clim.2003.11.017 15047203

[19] De Herdt V, Dumont F, Hénon H, Derambure P, Vonck K, Leys D, et al. Early seizures in intracerebral hemorrhage. Neurology. 2011;77(20):1794–800. doi: 10.1212/wnl.0b013e31823648a6 21975203

[20] De Reuck J, Hemelsoet D, Van Maele G. Seizures and epilepsy in patients with a spontaneous intracerebral haematoma. Clin Neurol Neurosurg. 2007;109(6):501–4. doi: 10.1016/j.clineuro.2007.04.001 17509750

[21] Bladin CF, Alexandrov AV, Bellavance A, Bornstein N, Chambers B, Coté R, et al. Seizures after stroke: a prospective multicenter study. Arch Neurol. 2000;57(11):1617–22. doi: 10.1001/archneur.57.11.1617 11074794

[22] Beghi E, D’Alessandro R, Beretta S, Consoli D, Crespi V, Delaj L, et al. Incidence and predictors of acute symptomatic seizures after stroke. Neurology. 2011;77(20):1785–93. doi: 10.1212/WNL.0b013e3182364878 21975208

[23] Qian C, Löppönen P, Tetri S, Huhtakangas J, Juvela S, Turtiainen H-ME, et al. Immediate, early and late seizures after primary intracerebral hemorrhage. Epilepsy Res. 2014;108(4):732–9. doi: 10.1016/j.eplepsyres.2014.02.020 24661429

[24] Li H, Prince DA. Synaptic activity in chronically injured, epileptogenic sensory-motor neocortex. J Neurophysiol. 2002;88(1):2–12. doi: 10.1152/jn.00507.2001 12091528

[25] Prince DA, Parada I, Scalise K, Graber K, Jin X, Shen F. Epilepsy following cortical injury: cellular and molecular mechanisms as targets for potential prophylaxis. Epilepsia. 2009;50 Suppl 2(Suppl 2):30–40. doi: 10.1111/j.1528-1167.2008.02008.x 19187292 PMC2710960

[26] Nicolo J-P, Chen Z, Moffat B, Wright DK, Sinclair B, Glarin R, et al. Study protocol for a phase II randomised, double-blind, placebo-controlled trial of perampanel as an antiepileptogenic treatment following acute stroke. BMJ Open. 2021;11(5):e043488. doi: 10.1136/bmjopen-2020-043488 33972334 PMC8112439

[27] Librizzi L, Vila Verde D, Colciaghi F, Deleo F, Regondi MC, Costanza M, et al. Peripheral blood mononuclear cell activation sustains seizure activity. Epilepsia. 2021;62(7):1715–28. doi: 10.1111/epi.16935 34061984

[28] Pernhorst K, Herms S, Hoffmann P, Cichon S, Schulz H, Sander T, et al. TLR4, ATF-3 and IL8 inflammation mediator expression correlates with seizure frequency in human epileptic brain tissue. Seizure. 2013;22(8):675–8. doi: 10.1016/j.seizure.2013.04.023 23706953

[29] Jia Q, Jiang F, Ma D, Li J, Wang F, Wang Z. Association Between IL-6 and Seizure Recurrence in Patients with the First Post-Ischemic Stroke Seizure. Neuropsychiatr Dis Treat. 2020;16:1955–63. doi: 10.2147/NDT.S257870 32848401 PMC7429209

[30] Cerri C, Genovesi S, Allegra M, Pistillo F, Püntener U, Guglielmotti A, et al. The Chemokine CCL2 Mediates the Seizure-Enhancing Effects of Systemic Inflammation. J Neurosci. 2016;36(13):3777–88. 27030762 10.1523/JNEUROSCI.0451-15.2016 PMC6601741

[31] Wu Y, Wang X, Mo X, Xi Z, Xiao F, Li J, et al. Expression of monocyte chemoattractant protein-1 in brain tissue of patients with intractable epilepsy. Clin Neuropathol. 2008;27(2):55–63. doi: 10.5414/npp27055 18402383

[32] Tian DS, Peng J, Murugan M, Feng LJ, Liu JL, Eyo UB. Chemokine CCL2–CCR2 signaling induces neuronal cell death via STAT3 activation and IL-1β production after status epilepticus. J Neurosci. 2017;37(33):7878–92. 28716963 10.1523/JNEUROSCI.0315-17.2017 PMC5559763

[33] Foresti ML, Arisi GM, Katki K, Montañez A, Sanchez RM, Shapiro LA. Chemokine CCL2 and its receptor CCR2 are increased in the hippocampus following pilocarpine-induced status epilepticus. J Neuroinflammation. 2009;6:40. doi: 10.1186/1742-2094-6-40 20034406 PMC2804573

[34] Česká K, Papež J, Ošlejšková H, Slabý O, Radová L, Loja T, et al. CCL2/MCP-1, interleukin-8, and fractalkine/CXC3CL1: Potential biomarkers of epileptogenesis and pharmacoresistance in childhood epilepsy. Eur J Paediatr Neurol. 2023;46:48–54. 37429062 10.1016/j.ejpn.2023.06.001

[35] Milano C, Montali M, Barachini S, Burzi IS, Pratesi F, Petrozzi L, et al. Increased production of inflammatory cytokines by circulating monocytes in mesial temporal lobe epilepsy: A possible role in drug resistance. J Neuroimmunol. 2024;386:578272. 38160122 10.1016/j.jneuroim.2023.578272

[36] Geng H, Chen L, Tang J, Chen Y, Wang L. The Role of CCL2/CCR2 Axis in Cerebral Ischemia-Reperfusion Injury and Treatment: From Animal Experiments to Clinical Trials. Int J Mol Sci. 2022;23(7):3485. doi: 10.3390/ijms23073485 35408846 PMC8998625

[37] Stowe AM, Wacker BK, Cravens PD, Perfater JL, Li MK, Hu R, et al. CCL2 upregulation triggers hypoxic preconditioning-induced protection from stroke. J Neuroinflammation. 2012;9:33. doi: 10.1186/1742-2094-9-33 22340958 PMC3298779

[38] Lee S, Kim OJ, Lee KO, Jung H, Oh S-H, Kim NK. Enhancing the Therapeutic Potential of CCL2-Overexpressing Mesenchymal Stem Cells in Acute Stroke. Int J Mol Sci. 2020;21(20):7795. doi: 10.3390/ijms21207795 33096826 PMC7588958

[39] Pimentel-Coelho PM, Michaud JP, Rivest S. C–C chemokine receptor type 2 (CCR2) signaling protects neonatal male mice with hypoxic–ischemic hippocampal damage from developing spatial learning deficits. Behav Brain Res. 2015;286:146–51. 25746456 10.1016/j.bbr.2015.02.053

[40] Da Silva-Candal A, Dopico-López A, Pérez-Mato M, Rodríguez-Yáñez M, Pumar JM, Ávila-Gómez P, et al. Characterization of a Temporal Profile of Biomarkers as an Index for Ischemic Stroke Onset Definition. J Clin Med. 2021;10(14):3136. doi: 10.3390/jcm10143136 34300300 PMC8307571

[41] Zafar A, Farooqui M, Ikram A, Suriya S, Kempuraj D, Khan M, et al. Cytokines, brain proteins, and growth factors in acute stroke patients: A pilot study. Surg Neurol Int. 2021;12:366. doi: 10.25259/SNI_569_2021 34513133 PMC8422456

[42] Luheshi NM, Kovács KJ, Lopez-Castejon G, Brough D, Denes A. Interleukin-1α expression precedes IL-1β after ischemic brain injury and is localised to areas of focal neuronal loss and penumbral tissues. J Neuroinflammation. 2011;8:186. doi: 10.1186/1742-2094-8-186 22206506 PMC3259068

[43] Wunderlich MT, Lins H, Skalej M, Wallesch C-W, Goertler M. Neuron-specific enolase and tau protein as neurobiochemical markers of neuronal damage are related to early clinical course and long-term outcome in acute ischemic stroke. Clin Neurol Neurosurg. 2006;108(6):558–63. doi: 10.1016/j.clineuro.2005.12.006 16457947

[44] Lekoubou A, Petucci J, Femi Ajala T, Katoch A, Hong J, Sen S, et al. Can machine learning predict late seizures after intracerebral hemorrhages? Evidence from real-world data. Epilepsy Behav. 2024;157:109835. doi: 10.1016/j.yebeh.2024.109835 38820686 PMC13280972

[45] Liu J, He H, Wang Y, Du J, Liang K, Xue J, et al. Predictive models for secondary epilepsy in patients with acute ischemic stroke within one year. eLife. 2024;13:RP98759. 10.7554/eLife.98759 PMC11563573 39540824

[46] Yu Y, Chen Z, Yang Y, Zhang J, Wang Y. Development and validation of an interpretable machine learning model for predicting post-stroke epilepsy. Epilepsy Res. 2024;205:107397. doi: 10.1016/j.eplepsyres.2024.107397 38976953

