# Supplementary material for: Characterizing inflammatory biomarkers in post-stroke seizure risk and outcome prognostication
Source: PLoS One. 2026 Mar 31;21(3):e0345752. doi: 10.1371/journal.pone.0345752 (PMC13038000; doi:10.1371/journal.pone.0345752)
Supplement: S1 File — (DOCX) [file pone.0345752.s001.docx]

**Table S1:** Paired sample t-test between biomarker values at 24 and 72 hours respectively.

| **Paired Samples Test** | | | | | |
| --- | --- | --- | --- | --- | --- |
|  | | Paired Differences | | | |
|  |  | Mean | Std. Deviation | Std. Error Mean | 95% Confidence Interval of the Difference |
|  |  |  |  |  | Lower |
| Pair 1 | CCL2 - 24h - CCL2 - 72h | -.933678397532049 | 125.388340173862830 | 20.340674736231513 | -42.147800241005420 |
| Pair 2 | GCSF - 24h - GCSF - 72h | 1.069651714360056 | 6.049475230803476 | .981354469036288 | -.918761314360936 |
| Pair 3 | IL10 - 24h - IL10 - 72h | -2.074794509890647 | 11.307416474998858 | 1.939205927079399 | -6.020138633437638 |
| Pair 4 | IL1b - 24h - IL1b - 72h | -.100088659433015 | 2.059444123910745 | .334085952520199 | -.777011098433253 |
| Pair 5 | IL6 - 24h - IL6 - 72h | 3.333590025385540 | 98.002158785895360 | 15.898049472130593 | -28.878917991905050 |
| Pair 6 | MIp1a - 24h - MIp1a - 72h | .002066781947473 | .541467125543348 | .092860845931865 | -.186860029634971 |
| Pair 7 | TNF - 24h - TNF - 72h | .316254150315794 | 4.896531022487670 | .839748142463038 | -1.392226291529717 |
| Pair 8 | IL8 - 24h - IL8 - 72h | -2.829206396039158 | 12.480716157412507 | 2.109626072499225 | -7.116482398588691 |

| **Paired Samples Test** | | | | | | |
| --- | --- | --- | --- | --- | --- | --- |
|  | | Paired Differences | t | df | Significance | |
|  |  | 95% Confidence Interval of the Difference |  |  | One-Sided p | Two-Sided p |
|  |  | Upper |  |  |  |  |
| Pair 1 | CCL2 - 24h - CCL2 - 72h | 40.280443445941320 | -.046 | 37 | .482 | .964 |
| Pair 2 | GCSF - 24h - GCSF - 72h | 3.058064743081048 | 1.090 | 37 | .141 | .283 |
| Pair 3 | IL10 - 24h - IL10 - 72h | 1.870549613656344 | -1.070 | 33 | .146 | .292 |
| Pair 4 | IL1b - 24h - IL1b - 72h | .576833779567223 | -.300 | 37 | .383 | .766 |
| Pair 5 | IL6 - 24h - IL6 - 72h | 35.546098042676130 | .210 | 37 | .418 | .835 |
| Pair 6 | MIp1a - 24h - MIp1a - 72h | .190993593529916 | .022 | 33 | .491 | .982 |
| Pair 7 | TNF - 24h - TNF - 72h | 2.024734592161306 | .377 | 33 | .354 | .709 |
| Pair 8 | IL8 - 24h - IL8 - 72h | 1.458069606510375 | -1.341 | 34 | .094 | .189 |

**Table S2:** Proportion of patients with samples collected at 24 hours, 72 hours or both timepoints.

|  | | 24 hour plasma | 72 hour plasma | Both | Sum |
| --- | --- | --- | --- | --- | --- |
| Early or Late | Early | 10 | 16 | 7 | 33 |
|  | Late | 6 | 15 | 8 | 29 |
|  | None | 51 | 36 | 23 | 110 |
| Total | | 67 | 67 | 38 | 172 |

**Note:** For late SED, no association was found between biomarkers and mortality or poor outcomes at any timepoint in univariate analyses. For both early SED and early and late SED combined, we were unable to find an association between biomarkers and poor outcomes at any timepoint in univariate analyses. These analyses are not included in tables below as a result.

**Table S3: Baseline Characteristics of Early SED by 90-Day Mortality**

| **Characteristics** | **90-Day Mortality**  **(N=13)** | **Non-90-Day Mortality (N=20)** | **p-value** |
| --- | --- | --- | --- |
| Demographics |  |  |  |
| Age | 70 (65-82) | 64 (56-70) | 0.043 |
| Male Gender | 6 (53.9) | 9 (45.0) | 0.948 |
| Caucasian | 9 (69.0) | 15 (75.0) | 0.716 |
| History of Smoking | 5 (38.5) | 10 (50.0) | 0.515 |
| Alcohol Use | 5 (38.5) | 8 (40.0) | 0.930 |
| Vascular Risk Factors |  |  |  |
| Hypertension | 8 (61.5) | 12 (60.0) | 0.930 |
| Diabetes | 5 (38.5) | 2 (10.0) | 0.051 |
| Hyperlipidemia | 7 (53.9) | 5 (25.0) | 0.092 |
| Atrial fibrillation | 1 (7.7) | 3 (15.0) | 0.530 |
| Coagulopathy | 1 (7.7) | 1 (5.0) | 0.751 |
| Coronary Artery Disease | 2 (15.4) | 1 (5.0) | 0.311 |
| Prior Transient Ischemic Attack | 0 (0) | 3 (15.0) | 0.143 |
| Prior Myocardial Infarction | 0 (0) | 0 (0) | N/A |
| Inpatient Antiplatelet Use | 0 (0) | 2 (10.0) | 0.239 |
| Clinical Scores |  |  |  |
| NIHSS at Admission | 20 (17-23) | 10 (5.5-19.5) | 0.004 |
| ICH Score | 2.0 (2.0-3.0) | 1.0 (0-2.5) | 0.028 |
| Radiological parameters |  |  |  |
| ICH volume | 35.1 (19.9-47.8) | 17.75 (5.05-40.7) | 0.077 |
| Lobar ICH | 8 (61.5) | 12 (60.0) | 0.930 |
| Biomarkers |  |  |  |
| CCL2 | 7.42 (6.79-8.45) | 7.35 (4.86-8.19) | 0.195 |
| IL6 | 5.36 (4.03-6.23) | 3.51 (2.58-5.38) | 0.077 |
| CXCL8 (IL8) | 4.23 (3.90-5.49) | 3.99 (3.16-4.54) | 0.063 |

All values are in Median (IQR) or n (%). p-values from Mann-Whitney U test, independent t-test, or χ^2^ test

**Table S4: Multivariable prediction model for 90-day mortality in Early SED**

| **Predictor Variables** | **OR (95% CI)** | **P-value** |
| --- | --- | --- |
| **Prediction Model: Early SED** | |  |
| Hyperlipidemia | 16.3 (1.10-240.24) | 0.042 |
| NIHSS at Admission | 1.33 (1.08-1.64) | 0.008 |

Model adjusted for ICH score, ICH volume, NIHSS at admission, diabetes, hyperlipidemia, IL6, CXCL8 (IL8)

**Table S5: Baseline Characteristics of Early SED by 180-Day Mortality**

| **Characteristics** | **180-Day Mortality**  **(N=17)** | **Non-180-Day Mortality (N=16)** | **p-value** |
| --- | --- | --- | --- |
| Demographics |  |  |  |
| Age | 69 (65-79) | 62 (55-70) | 0.038 |
| Male Gender | 8 (47.1) | 7 (43.8) | 0.849 |
| Caucasian | 12 (70.6) | 12 (75.0) | 0.776 |
| History of Smoking | 8 (47.1) | 7 (43.8) | 0.849 |
| Alcohol Use | 7 (41.2) | 6 (37.5) | 0.829 |
| Vascular Risk Factors |  |  |  |
| Hypertension | 11 (64.7) | 9 (56.3) | 0.619 |
| Diabetes | 6 (35.3) | 1 (6.3) | 0.041 |
| Hyperlipidemia | 8 (47.1) | 4 (25.0) | 0.188 |
| Atrial fibrillation | 1 (5.9) | 3 (18.8) | 0.258 |
| Coagulopathy | 1 (5.9) | 1 (6.3) | 0.965 |
| Coronary Artery Disease | 3 (17.7) | 0 (0) | 0.078 |
| Prior Transient Ischemic Attack | 2 (11.8) | 1 (6.3) | 0.582 |
| Prior Myocardial Infarction | 0 (0) | 0 (0) | N/A |
| Inpatient Antiplatelet Use | 2 (11.8) | 0 (0) | 0.157 |
| Clinical Scores |  |  |  |
| NIHSS at Admission | 20 (17-23) | 9 (5-17) | 0.002 |
| ICH Score | 2 (2-3) | 1 (0-2) | 0.008 |
| Radiological parameters |  |  |  |
| ICH volume | 34 (18.6-47.8) | 17.8 (5.1-40.7) | 0.140 |
| Lobar ICH | 10 (58.8) | 10 (62.5) | 0.829 |
| Biomarkers |  |  |  |
| CCL2 | 7.2 (6.3-8.1) | 7.4 (6.1-8.4) | 0.858 |
| IL6 | 4.5 (2.8-6.1) | 3.9 (2.7-6.0) | 0.525 |
| CXCL8 (IL8) | 4.1 (3.9-5.5) | 4.1 (3.6-4.7) | 0.091 |

All values are in Median (IQR) or n (%). p-values from Mann-Whitney U test, independent t-test, or χ^2^ test

**Table S6: Multivariable prediction model for 180-day mortality in Early SED**

| **Predictor Variables** | **OR (95% CI)** | **P-value** |
| --- | --- | --- |
| **Prediction Model: Early SED** | |  |
| Age | 1.13 (1.00-1.27) | 0.046 |
| NIHSS at Admission | 1.30 (1.07-1.57) | 0.008 |

Model adjusted for NIHSS at admission, IL6, CXCL8 (IL8), age, ICH score, diabetes

**Table S7: Baseline Characteristics of Early and Late SED (Combined) by 180-Day Mortality**

| **Characteristics** | **180-Day Mortality**  **(N=21)** | **Non-180-Day Mortality (N=41)** | **p-value** |
| --- | --- | --- | --- |
| Demographics |  |  |  |
| Age | 72 (67-79) | 65 (53-74) | 0.017 |
| Male Gender | 9 (42.9) | 20 (48.8) | 0.658 |
| Caucasian | 15 (71.4) | 29 (70.7) | 0.954 |
| History of Smoking | 9 (42.9) | 17 (41.5) | 0.916 |
| Alcohol Use | 7 (33.3) | 12 (29.3) | 0.742 |
| Vascular Risk Factors |  |  |  |
| Hypertension | 12 (57.1) | 25 (61.0) | 0.771 |
| Diabetes | 6 (28.6) | 1 (2.4) | 0.002 |
| Hyperlipidemia | 11 (52.4) | 15 (36.6) | 0.233 |
| Atrial fibrillation | 3 (14.3) | 7 (17.1) | 0.778 |
| Coagulopathy | 1 (4.8) | 2 (4.9) | 0.984 |
| Coronary Artery Disease | 3 (14.3) | 3 (7.3) | 0.380 |
| Prior Transient Ischemic Attack | 3 (14.3) | 2 (4.9) | 0.198 |
| Prior Myocardial Infarction | 0 (0) | 1 (2.4) | 0.471 |
| Inpatient Antiplatelet Use | 2 (9.5) | 4 (9.8) | 0.977 |
| Clinical Scores |  |  |  |
| NIHSS at Admission | 20 (15-23) | 8 (4-16) | <0.0001 |
| ICH Score | 2.0 (1.0-3.0) | 1.0 (0.0-2.0) | 0.0002 |
| Radiological parameters |  |  |  |
| ICH volume | 35.1 (18.6-49.7) | 15.3 (8.1-31.6) | 0.022 |
| Lobar ICH | 13 (61.9) | 29 (70.7) | 0.482 |
| Biomarkers |  |  |  |
| CCL2 | 7.11 (5.17-8.01) | 6.31 (4.77-7.93) | 0.339 |
| IL6 | 4.52 (2.80-5.66) | 3.98 (2.54-5.24) | 0.320 |
| CXCL8 (IL8) | 4.23 (3.90-5.46) | 3.64 (2.94-4.55) | 0.017 |

All values are in Median (IQR) or n (%). p-values from Mann-Whitney U test, independent t-test, or χ^2^ test

**Table S8: Multivariable prediction model for 180-day mortality in Early and Late SED (Combined)**

| **Predictor Variables** | **OR (95% CI)** | **P-value** |
| --- | --- | --- |
| **Prediction Model: Early SED + Late SED** | |  |
| Age | 1.08 (1.01-1.16) | 0.020 |
| NIHSS at Admission | 1.31 (1.13-1.52) | <0.0001 |

Model adjusted for ICH score, ICH volume, NIHSS admission, diabetes, CXCL8 (IL8)

**Table S9: Baseline Characteristics of Early and Late SED (Combined) by 365-Day Mortality**

| **Characteristics** | **365-Day Mortality**  **(N=23)** | **Non-365-Day Mortality (N=39)** | **p-value** |
| --- | --- | --- | --- |
| Demographics |  |  |  |
| Age | 73 (67-79) | 64 (53-74) | 0.005 |
| Male Gender | 10 (43.5) | 19 (48.7) | 0.690 |
| Caucasian | 17 (73.9) | 27 (69.2) | 0.695 |
| History of Smoking | 9 (39.1) | 17 (43.6) | 0.731 |
| Alcohol Use | 7 (30.4) | 12 (30.8) | 0.978 |
| Vascular Risk Factors |  |  |  |
| Hypertension | 14 (60.9) | 23 (60.0) | 0.883 |
| Diabetes | 6 (26.1) | 1 (2.6) | 0.005 |
| Hyperlipidemia | 12 (52.2) | 14 (35.9) | 0.210 |
| Atrial fibrillation | 4 (17.4) | 6 (15.4) | 0.836 |
| Coagulopathy | 1 (4.4) | 2 (5.1) | 0.890 |
| Coronary Artery Disease | 3 (13.0) | 3 (7.7) | 0.491 |
| Prior Transient Ischemic Attack | 3 (13.0) | 2 (5.1) | 0.269 |
| Prior Myocardial Infarction | 0 (0) | 1 (2.6) | 0.439 |
| Inpatient Antiplatelet Use | 3 (13.04) | 3 (7.7) | 0.491 |
| Clinical Scores |  |  |  |
| NIHSS at Admission | 20 (15-23) | 8 (4-16) | 0.0001 |
| ICH Score | 2.0 (1.0-3.0) | 1.0 (0.0-2.0) | 0.0005 |
| Radiological parameters |  |  |  |
| ICH volume | 34.0 (10.5-49.7) | 20 (8.1-35.6) | 0.092 |
| Lobar ICH | 14 (60.9) | 28 (71.8) | 0.374 |
| Biomarkers |  |  |  |
| CCL2 | 7.16 (5.10-8.09) | 6.54 (4.74-7.94) | 0.341 |
| IL6 | 4.65 (2.72-5.66) | 3.88 (2.54-5.33) | 0.475 |
| CXCL8 (IL8) | 4.18 (3.61-5.46) | 3.44 (2.85-4.39) | 0.027 |

All values are in Median (IQR) or n (%). p-values from Mann-Whitney U test, independent t-test, or χ^2^ test

**Table S10: Multivariable prediction model for 365-day mortality in Early and Late SED (Combined)**

| **Predictor Variables** | **OR (95% CI)** | **P-value** |
| --- | --- | --- |
| **Prediction Model: Early SED + Late SED** | |  |
| Age | 1.08 (1.02-1.15) | 0.012 |
| NIHSS at Admission | 1.25 (1.11-1.42) | <0.0001 |

Model adjusted for ICH score, ICH volume, NIHSS admission, diabetes, CXCL8 (IL8)
